# Torticollis in Non-Syndromic Unicoronal Craniosynostosis Is Predominantly Ocular Related

**DOI:** 10.3390/jcm12186059

**Published:** 2023-09-19

**Authors:** Emily T. C. Tan, Parinaz Rostamzad, Yasmin S. Esser, Mieke M. Pleumeekers, Sjoukje E. Loudon

**Affiliations:** 1 Department of Ophthalmology, Erasmus MC, University Medical Center, 3000 CA Rotterdam, The Netherlands; 2 Clinical Sciences for Health Professionals, Program in Clinical Health Sciences, University Medical Center Utrecht, Utrecht University, 3584 CS Utrecht, The Netherlands; 3 Department of Plastic and Reconstructive Surgery, Erasmus MC, University Medical Center, 3000 CA Rotterdam, The Netherlands

**Keywords:** non-syndromic unicoronal craniosynostosis, torticollis, prevalence, ophthalmic features, plagiocephaly

## Abstract

(1) Background: Patients with unicoronal craniosynostosis (UCS) often show torticollis which can result from either an ocular cause or contraction of the sternocleidomastoid muscle. For clinicians, it is crucial to know the prevalence of ocular torticollis (OT) to ensure appropriate referral for treatment. Furthermore, associated ophthalmic features with OT in these patients are scarcely described. The aim of this study was to determine the prevalence of OT in non-syndromic UCS patients and investigate its associated ophthalmic features. (2) Methods: In this descriptive cross-sectional study medical records of non-syndromic UCS patients treated between 1994–2022 in one tertiary care hospital in The Netherlands were retrospectively reviewed. Collected data included: diagnosis and type of torticollis, binocular single vision (BSV), strabismus, ocular motility, alphabetical patterns, refractive error, and amblyopia. Patients were classified as OT, based on their ophthalmic and/or orthoptic diagnosis. Prevalence was determined with the 95% CI using the Clopper–Pearson exact test. Associations between OT and the ophthalmic features were determined using Chi-square or Fishers’ exact test and its effect size was calculated using Cramer’s V. (3) Results: In total, 146 patients were included, of whom 57 had torticollis. An ocular cause for the torticollis was found in 54 patients. The prevalence of OT was 37% (n = 146; 95% CI [0.292–0.454]). Significant associations were found between OT and strabismus (*p* < 0.001), ocular motility abnormalities (*p* < 0.001), alphabetical patterns (*p* < 0.001), and amblyopia (*p* = 0.002). BSV (*p* = 0.277) and refractive error (*p* = 1.0) were not significantly associated with OT. However, in OT the BSV was relatively poor (42.1%) and more frequently absent (26.3%) compared to the non-torticollis group (7% poor and 16.3% absent). In both groups, excyclotorsion was predominantly present (62.3%). (4) Conclusions: In 95% of cases, torticollis in UCS patients is ocular-related. Overall, one in three patients with UCS have OT. This study emphasizes the importance of a timely referral of all patients with UCS with torticollis to an orthoptist and/or ophthalmologist, specialized in diagnosing and treatment of OT, before considering physiotherapy.

## 1. Introduction

Craniosynostosis is a rare congenital condition characterized by the premature closure of one or more cranial sutures [1]. It can occur as an isolated finding (non-syndromic) or as part of a syndrome [2,3]. In non-syndromic unicoronal craniosynostosis (UCS) one coronal suture is prematurely closed [3,4]. It is also known as anterior plagiocephaly and accounts for 30–35% of non-syndromic craniosynostosis cases [5]. The prevalence of UCS is approximately 1 in 10,000 live births worldwide, with a higher incidence in females [4,6]. The premature closure of the coronal sutures results in several skull deformations: retrusion of the forehead and superior orbital rim, contralateral frontal bossing of the forehead, and orbital asymmetry [7]. Ocular abnormalities associated with UCS include strabismus, aniso-astigmatism, and amblyopia as a result of craniofacial malformation/deformity [8]. Additionally, torticollis can occur in UCS, which can be characterized as ocular-related or non-ocular-related torticollis [9,10,11,12].

So far, the prevalence of ocular torticollis (OT) in patients with UCS remains unknown, despite being a commonly reported feature [11,13]. Determining this prevalence is crucial for clinicians, to make appropriate referrals for treating torticollis. Non-ocular torticollis in UCS can have multiple causes, including that as a result of sustained contraction of the sternocleidomastoid muscle or cervical vertebral segmentation anomalies [14,15,16,17]. In these cases, physiotherapy is needed to prevent permanent torticollis by relieving tense neck muscles and achieving an upright head position [9,10,12,18]. On the contrary, OT is an abnormal head posture resulting from an ocular cause. It serves as a compensatory mechanism to alleviate double vision caused by strabismus and obtain binocular single vision (BSV) or to improve visual acuity (VA) [19]. Maintaining OT is crucial for preserving BSV [19,20,21,22]. If OT is not maintained, it can lead to diplopia, suppression and the development of amblyopia [22,23]. Referral to an orthoptist and ophthalmologist is essential for treating OT, as their expertise is to effectively resolve OT through strabismus surgery [20,23].

A significantly higher prevalence of strabismus is found in UCS patients [24,25,26,27,28,29,30] compared to the general population [31]. Strabismus in UCS can result from the abnormal position of the trochlea or the absence of the superior oblique muscle [27,30]. This can lead to excyclotorsion syndrome, also known as pseudo-superior oblique palsy or strabismus sursoadductorius [32,33]. The excyclotorsion syndrome causes several ocular motility abnormalities to occur: an elevation in adduction, V-pattern strabismus, and excyclotorsion [11,34]. The high prevalence of strabismus is hypothesized as the ocular reason why these patients adopt torticollis [11,34]. So far, these associated ophthalmic features in UCS patients with OT are scarcely described [11,35,36].

The primary aim of this study is to determine the prevalence of OT in patients diagnosed with non-syndromic UCS. Second, this study aims to investigate the association between the presence of OT and BSV, strabismus, ocular motility, presence of cyclotorsion, refractive error and amblyopia among these patients. This information is necessary for clinicians to make appropriate referrals for treating torticollis in UCS patients, to either an orthoptist and/or ophthalmologist or physiotherapist.

## 2. Materials and Methods

### 2.1. Study Design and Participants

In this retrospective descriptive population-based cross-sectional study, the medical records of patients with non-syndromic UCS treated between 1994 and 2022 at a tertiary-care hospital (Erasmus MC, Rotterdam, The Netherlands) were reviewed. This is the designated center for craniofacial surgery in The Netherlands. Therefore, the sample of patients is representative of the population of interest. Patients eligible for inclusion were those who had received a diagnosis of non-syndromic UCS, confirmed through CT scans evaluated by a radiologist and medical examination(s). Also, the non-syndromic form of UCS was confirmed with genetic testing in eligible patients. Patients were included if surgical, ophthalmologic, and/or orthoptic assessments were available. Patients with any syndromic craniofacial disorders were excluded. Also, patients with a history of strabismus surgery elsewhere were excluded from the study, as this could potentially affect or resolve an observed torticollis in these patients. Patients were categorized into two groups to compare their ophthalmic features in relation to OT: UCS patients with OT and those without. Patients with non-ocular torticollis were included in the group without torticollis.

### 2.2. Variables

The collected information for each patient included their sex, side of synostosis, type and age at craniofacial surgery, and age at the initial visit. Clinical data from the initial ophthalmologic and/or orthoptic visit were collected, which included the following: (1) presence or absence of observed torticollis and its type; (2) BSV; (3) presence of strabismus; (4) ocular motility; (5) presence or absence of cyclotorsion; (6) refractive error; and (7) presence or history of amblyopia. All clinical data, except for cyclotorsion, were obtained from the initial visit. If cyclotorsion was not determined during the initial visit, the first determination prior to strabismus surgery was considered. To ensure the quality and accuracy of the final dataset used in the analysis, excluded patients were discussed by two researchers (ET and PR). Any ambiguities or uncertainties were resolved through discussion within the team (ET, PR, and SL). Incomplete examinations were included as missing data.

### 2.3. Orthoptic Measurements

The variables were determined based on the orthoptic measurements as described below:

(1) The presence of torticollis was collected by reviewing the records of the orthoptist and ophthalmologist for the description of observed torticollis. Also, the records of the Department of Plastic and Reconstructive Surgery were scanned for descriptions of torticollis. The type of torticollis was defined as a face turn, chin elevation, chin depression, head tilt or a combination of the aforementioned. Patients were considered to have OT based on their ophthalmic and/or orthoptic diagnosis.
(2) The BSV was determined using the following stereotests: Bagolini Straited Glasses (positive or negative outcome). Or, the Lang, Titmus housefly, and/or TNO tests (outcomes measured in arc seconds). Outcomes were categorized as ‘not present’ if Bagolini was negative, ‘poor’ if Bagolini was positive and Titmus housefly measured 3.552 arc seconds; ‘moderate’ if Titmus circles 200–40 arc seconds were recognized; and ‘good’ if all figures of the Lang Test (200 arc seconds) or TNO plate V (480–240 arc seconds), VI or VII (120–15 arc seconds) were positive [37].
(3) The angle of the strabismus (horizontal, vertical, or combined deviations) was measured using the prism cover test at nearly (30 cm) in prism degrees. Outcomes were categorized as esotropia, exotropia, esotropia and vertical deviation, exotropia and vertical deviation, vertical deviation alone, or not present.
(4) Ocular motility abnormalities were described as elevation or depression in the adduction of one or both eyes, if present. The presence of an alphabetical pattern, defined as a V- or A-strabismus pattern or not present, was also noted during the assessment of ocular motility.
(5) Cyclotorsion was measured using the Maddox Double Rod test or assessed anatomically by the ophthalmologist during fundus examination. Outcomes were defined as incyclotorsion, excyclotorsion, or not present.
(6) Refractive errors were obtained by retinoscopy in cycloplegia. Outcomes were defined as hypermetropia (≥+1.0 D), high hypermetropia (≥+5.0 D), myopia (≥−1.0 D), high myopia (≥−5.0 D), astigmatism (≥−1.0 D) and anisometropia (≥1.0 D).
(7) The presence of amblyopia was defined as a visual acuity (VA) difference of ≥0.2 LogMAR between both eyes and was categorized as present or not present. VA was measured using the Lea Hyvarine, Amsterdam Picture Chart, tumbling E-chart, or Snellen chart depending on the age of the patient.

### 2.4. Statistical Methods

Descriptive statistics were analyzed using SPSS V.26 (IBM Corps, Armonk, NY, USA). The prevalence of torticollis in patients with UCS was determined using the Clopper–Pearson exact test with a 95% CI. To characterize the ophthalmic features of UCS patients with and without OT, descriptive statistics were conducted. The independent samples *t*-test was used to compare means between groups. Levene’s Test was used to test for homogeneity of variance. The results of each qualitative variable were presented by frequency (n, %). If the expected frequency in each cell of the contingency table was ≥5, a chi-square test was used to verify the association. If the expected frequency in each cell was <5 in >20% of cells, a Fisher’s exact test was used. Also, Cramer’s V test was performed to calculate the effect size for both the chi-square and Fisher’s exact test. Depending on the degrees of freedom, the effect size was rated as small, moderate, or strong [38]. Significant associations underwent further analysis to identify key contributing features to torticollis using a logistic regression model for variables with a binary outcome. In all analyses, *p* < 0.05 was considered statistically significant.

### 2.5. Reporting and Ethical Issues

The Strengthening the Reporting of Observational Studies in Epidemiology Statement (STROBE) for cross-sectional studies was used as a transparency reporting guideline. The study was conducted in accordance with the principles of the Declaration of Helsinki (2013). Since it was part of a larger observational longitudinal study approved by the Erasmus MC’s ethical board (MEC-2022-0367), this study was not subject to the Medical Research Involving Human Subjects Act (WMO).

## 3. Results

### 3.1. Study Characteristics and Participants

**Figure 1:**
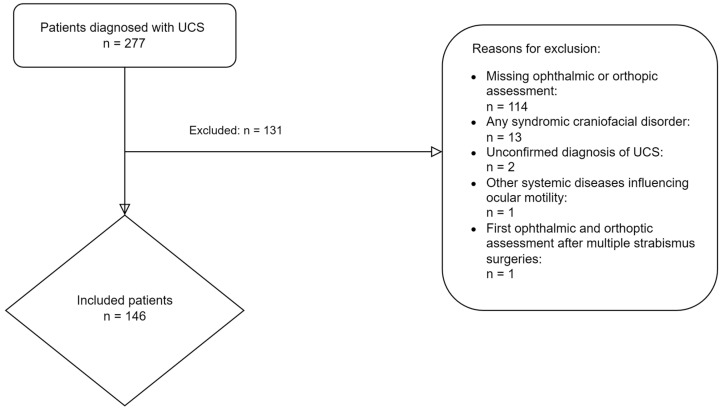
Flowchart of included patients and reasons for exclusion. Abbreviations: UCS: unicoronal craniosynostosis.

A total of 277 children diagnosed with UCS were treated at the Erasmus MC; 146 met the inclusion criteria. Most patients (n = 114) were excluded due to missing orthoptic assessments, any syndromic craniofacial disorder (n = 13), an unconfirmed diagnosis of UCS (n = 2), the presence of Graves’ Orbitopathy which could influence the ocular motility (n = 1) or a history of strabismus surgery (n = 1) (Figure 1).

Of the included patients 96 (65.8%) were female. The right coronal suture was closed in 85 (58.2%) patients. The mean age at the initial visit was 2.72 years (SD 3.6, range 0–27 years). No significant differences were observed in patients’ characteristics between patients with and without torticollis (Table 1).

### 3.2. The Prevalence of Torticollis 

Of the 146 patients identified with UCS, 57 patients (39%) had torticollis. Of the 57 patients identified with torticollis, 94.7% (n = 54) were ocular related. The prevalence of OT in all patients was found to be 37% (95% CI [0.292–0.454]). The mean age of consultation at which torticollis was identified was 2.8 years (SD 2.6, range 9 months-12 years). The most common diagnosis for OT was strabismus (93%, n = 53), of which 88.7% was incomitant (Table 2). The majority showed a pseudo-superior oblique palsy (59.6%), followed by pseudo-inferior oblique palsy (12.3%), accommodative esotropia (5.3%), and infantile esotropia combined with pseudo-superior oblique palsy (5.3%). The type of torticollis was specified in 56 patients: a head tilt was present in 47.4%, a face turn in 28.1%, or a combination in 22.8% (Table 3). Torticollis with only a chin elevation or depression was not present.

### 3.3. The Ophthalmic-Related Features Associated with OT

**Figure 2:**
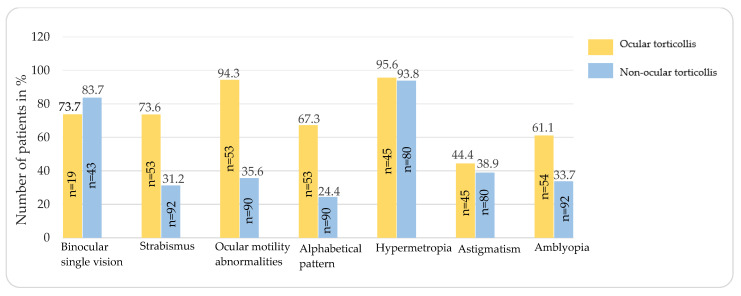
The presence of ophthalmic features in the ocular torticollis group and non-ocular torticollis group.

Collected ophthalmic-related features and their frequencies are shown in Table 4. Table 5 compares the occurrence of these features in UCS patients with or without OT, their association with the presence of OT, and the effect size (V) (Figure 2).

#### 3.3.1. Binocular Single Vision

BSV was determined in 19/54 (35.2%) patients with OT and 43/92 (46.7%) patients without (ocular) torticollis. In the OT group, BSV was present in 73.7% of patients (n = 14) compared to 83.7% (n = 36) of patients in the non-torticollis (NT) group. There was no significant association between BSV and OT presence (*p* = 0.277) (Table 5). In the OT group, BSV was relatively poor (n = 8, 42.1%) and more frequently absent (n = 5, 26.3%) compared to the NT group (n = 3, 7% and n = 7, 16.3%, respectively) (Table 4). 

#### 3.3.2. Strabismus

In 53 (98%) patients with OT and 92 (100%) patients without (ocular) torticollis, the presence of strabismus was assessed. In the OT group, strabismus was observed in 73.6% (n = 39) of patients, compared to 31.2% (n = 29) in the NT group. The association between strabismus and OT was moderate (V = 0.406) and statistically significant (χ_2_(1, n = 145) = 23.891, *p* < 0.001) (Table 5). In the OT group, a vertical deviation alone was predominantly present (24.5%, n = 13), followed by a combination of exotropia and vertical deviation (20.8%, n = 11), and a combination of esotropia and vertical deviation (18.9%, n = 10). These frequencies were higher compared to the non-OT group (4.3% n = 4, 3.3% n = 3, and 9.8% n = 9 respectively) (Table 4).

A vertical deviation (alone and combined with a horizontal deviation) was present in 64.2% (n = 34) of patients in the OT group, compared to 17.4% (n = 16) of patients in the NT group. A hypertropia was most frequently present in both groups, with a higher occurrence in the OT group (90.3%, n = 28) than in the NT group (76.4%, n = 13). In both groups, the hypertropic eye was 89.3% on the side of the fused suture in the OT group and 92.3% in the NT group. 

#### 3.3.3. Ocular Motility

Ocular motility was assessed in 53 (98%) patients with OT and 90 (97.8%) patients without (ocular) torticollis. A strong association (V = 0.574) was found between abnormalities in ocular motility and OT (χ_2_(1, n = 143) = 47.123, *p* < 0.001) (Table 5). Elevation in adduction in one or both eyes was the most frequently observed abnormality in both groups: one eye in 55.3% (n = 27) in the OT group and 21.1% (n = 19) in the NT group, and both eyes in 26.4% (n = 14) in the OT group and 10% (n = 9) in the NT group. In 55% of patients, the elevation in adduction was on the side of the fused suture. 

#### 3.3.4. Alphabetical Pattern 

A significant association was found between alphabetical patterns and OT (χ_2_(1, n = 143) = 26.157, *p* < 0.01) with a moderate effect size (V = 0.428) (Table 5). In the OT group, 54.7% (n = 29) of patients showed a V-pattern and 13.2% (n = 7) of patients showed an A-pattern. In contrast, in the NT group, 18.9% (n = 17) of patients showed a V-pattern and 5.6% (n = 5) of patients had an A-pattern (Table 4).

#### 3.3.5. Cyclotorsion

In total, the cyclotorsion was determined in 53 patients: 30/54 (55%) patients with OT, and 23/92 (25%) patients without (ocular) torticollis. An ophthalmologist anatomically determined cyclotorsion in most cases (52.8%, n = 28). In the OT group, excyclotorsion was observed in the majority of patients (73.3%, n = 22), while only 1 patient (3.3%) showed incyclotorsion. In the NT group, excyclotorsion was also most common (47.8%, n = 11), with only 3 patients (13%, n = 3) showing incyclotorsion (Table 4). Since the cyclotorsion in the NT group was only determined when abnormalities of the motility were observed or prior to strabismus surgery, no valid comparisons could be made between the two groups and no association could be assessed.

#### 3.3.6. Association between the Presence of Strabismus, Ocular Motility Abnormalities and Alphabetical Patterns

When determining the associations for both groups together (n = 146), significant associations were found between the presence of strabismus and ocular motility abnormalities (χ_2_(1, n = 142) = 33.157, *p* < 0.01), strabismus and alphabetical patterns (χ_2_(1, n = 142) = 24.521, *p* < 0.01), as well as between ocular motility abnormalities and alphabetical patterns (χ_2_(1, n = 143) = 56.051, *p* < 0.01) (Table 6). So, a logistic regression model could not be conducted due to multicollinearity.

#### 3.3.7. Refractive Error 

The refractive error was obtained in 125 patients: 45/54 (83.3%) patients with OT and 80/92 (87%) patients without (ocular) torticollis. In both the OT group and NT group, hypermetropia was most prevalent (89.1% n = 41 and 89.9% n = 71, respectively), followed by astigmatism (43.5% n = 20 and 44.3% n = 35, respectively) and anisometropia (26.7% n = 12 and 26.3% n = 21, respectively) (Table 4). No significant associations were present between any type of refractive error and OT (*p* = 1.0) (Table 5).

#### 3.3.8. Amblyopia 

Amblyopia was present in 33 (61%) patients with OT and 31 (33.7%) patients without (ocular) torticollis (Table 4). A significant association was found between OT and the presence of amblyopia (χ_2_(1, n = 146) = 10.38, *p* = 0.002), although its effect size was small (V = 0.267) (Table 5).

## 4. Discussion

This study presents the largest cohort of non-syndromic UCS patients to determine the prevalence of OT and its associated ophthalmic features. In 95% of cases, torticollis in UCS patients is ocular related. Overall, one in three patients with UCS have OT. A significant association was found between the presence of OT and strabismus, ocular motility abnormalities, alphabetical patterns, and amblyopia. UCS patients with OT had a relatively poorer BSV compared to those without torticollis. An excyclotorsion of one or both eyes was present in half of the patients. The findings of this study imply that in patients with non-syndromic UCS, torticollis is highly associated with an underlying ocular cause. 

Strabismus is the leading cause of OT (80%) in the general population, with incomitant strabismus as the most frequent form (80%) [39,40,41,42]. Nystagmus is found as the second leading cause [18,39,40,41,42,43] followed by infantile esotropia [39,41]. The present study, only including patients with UCS, yielded similar results: strabismus accounted for the majority (82%) of OT cases, with incomitant strabismus (88.7%) being the most prevalent identifiable cause. Only one patient (1.8%) had OT caused by congenital nystagmus. Earlier studies have reported a prevalence of strabismus in UCS patients ranging from 40–70% [24,25,26,27,28,29]. An incomitant type of strabismus [28,35,36,44] was more frequently observed than a concomitant type of strabismus in patients with UCS [29]. In the present study, a similar occurrence of strabismus was found (47.2%), mostly incomitant. However, within the study groups, the prevalence of strabismus was higher in the OT group (73.3%) and lower in the NT group (31.2%). The present study’s findings indicate that similarly to the general population, (incomitant) strabismus is the primary cause of OT in patients with UCS.

A reason for adopting OT in (incomitant) strabismus is to achieve BSV, by adjusting their head posture in the gaze direction where the angle of strabismus is least pronounced [19,20,21,22]. Surprisingly, no significant association was found between BSV and OT in patients with UCS in this study. Hence, a strong correlation was observed between OT and ocular motility abnormalities, and a moderate association regarding alphabetical patterns. The observed ocular motility abnormalities and alphabetical patterns are consistent with previous studies. Touzé et al. [11,34] demonstrated similar findings in UCS patients: an elevation in adduction was present in 65% (n = 13) of patients and V-pattern strabismus was present in 45% (n = 9) of patients. MacIntosh et al. [28] demonstrated an elevation in adduction in half of their included UCS patients. These findings suggest that UCS patients adopt OT in order to overcome ocular motility abnormalities and/or alphabetical patterns in (incomitant) strabismus, and not solely for obtaining BSV.

Ocular manifestations like strabismus, ocular motility abnormalities, cyclotorsion, and amblyopia in UCS are believed to result from various mechanical processes. UCS patients typically exhibit structural differences, including a taller, narrower and smaller ipsilateral orbit compared to the vertically shorter, wider and larger contralateral orbit [45,46]. This anatomical variation leads to a shorter and weaker ipsilateral superior oblique muscle [45]. Furthermore, displacement or absence of the superior oblique muscle has been observed [47]. These factors create an imbalance between the inferior- and superior oblique muscles, resulting in a pseudo-superior oblique palsy. Consequently, the inferior oblique muscle overcompensates excessively, leading to vertical manifest strabismus ipsilateral to the side of the synostosis, elevation in adduction, the presence of a V-pattern, excyclotorsion of the eye [30,48] and amblyopia [35,36]. The present study confirms these ophthalmic features in UCS patients and indicates their heightened severity in those with OT. Although a direct association between the presence of OT and cyclotorsion could not be established, excyclotorsion was most commonly observed in both groups. An alternative reason for these more severe abnormal manifestations in UCS patients with OT could be the relatively poorer and more frequent absence of BSV. Previous research supports this hypothesis, demonstrating a strong correlation between the absence of BSV and the occurrence of elevation adduction, V pattern, and excyclotorsion, all of which increased when BSV was absent [49].

Torticollis in patients with UCS is predominantly ocular-related, as demonstrated by the findings of this study, which is important knowledge for clinicians treating these patients. It highlights the importance of timely referral of all UCS patients with torticollis to an orthoptist and/or ophthalmologist for screening before considering physiotherapy. Orthoptists and ophthalmologists are experts in diagnosing OT and identifying the associated ophthalmic features found in these patients, including strabismus, ocular motility abnormalities, alphabetical patterns, amblyopia and relatively poorer BSV. Orthoptists can effectively treat OT by using glasses or strabismus surgery [20,23,50]. Timely treatment of OT can prevent complications associated with permanent torticollis including neck pain, headache, muscular and soft tissue changes, facial asymmetry, and scoliosis [20,43,50,51].

### Strengths and Limitations

The current study’s strength is its substantial sample size of 146 patients, exceeding previous studies on ophthalmic features in UCS patients ranging from 15 to a maximum of 59 included patients [11,28,34,35,36,39]. Furthermore, this study is the first to differentiate between the presence of these features in cases with OT and those without (ocular) torticollis. However, a limitation of this retrospective study is the absence of systematic examinations, potentially introducing information bias. The data used in this study was obtained from routine ophthalmic and orthoptic care, lacking protocolized assessments. In routine care, the presence of manifest strabismus often leads to the assumption of absent BSV resulting in a limited number of patients with BSV measurements. Similarly, determining cyclotorsion in patients without torticollis was missing, as it was primarily assessed in patients preparing for strabismus surgery. Therefore, no valid association between OT and cyclotorsion could be established. We assume that if cyclotorsion was determined in all UCS patients without torticollis, an association between the presence of OT and cyclotorsion would be found. The reported prevalence of OT in this study is conducted in a population that was referred for ophthalmology evaluation specifically due to the presence of torticollis. Therefore, OT could be overrepresented. To address this potential bias, the records of the Department of Plastic and Reconstructive Surgery were examined to determine if there were cases of torticollis that were not referred to the Department of Ophthalmology for further evaluation of its cause. However, the number of such cases was extremely small, indicating that the presented prevalence of OT is likely to be reliable. Future studies should consider larger prospective multicenter studies, also including other forms of synostosis creating asymmetry of the skull like unilamboid and unifrontosphenoid synostose, with standardized ophthalmological and orthoptic assessments, as well as long-term follow-up evaluations. Additionally, 3D studies analyzing soft tissue changes and facial asymmetry in OT in UCS patients and its implications should be investigated.

## 5. Conclusions

Torticollis in patients with non-syndromic UCS is predominantly ocular-related. Overall, one in three patients with UCS have OT. This makes it essential for clinicians to refer all UCS patients with torticollis to an orthoptist and/or ophthalmologist who possesses the expertise to diagnose and treat OT and its related ophthalmic features. The study’s findings suggest a need to reconsider current guidelines for ophthalmic care in UCS patients, placing emphasis on referring all UCS patients with torticollis to an orthoptist and/or ophthalmologist for screening before considering physiotherapy.

## Figures and Tables

**Table 1:** Patient characteristics of included UCS patients (n = 146).

Patient Characteristics	Total (n = 146)	Torticollis (n = 57)	No Torticollis (n = 89)	*p*-Value
Sex (n, %)				0.853
Female	96 (65.8)	38 (66.7)	58 (65.2)	
Male	50 (44.2)	19 (33.3)	31 (34.8)	
Synostotic coronal suture (n, %)				0.428
Right	85 (58.2)	31 (54.4)	54 (60.7)	
Left	58 (39.7)	25 (43.9)	33 (37.1)	
Unknown	3 (2.1)	1 (1.8)	2 (2.2)	
Age at initial visit (y, mean (SD))	2.72 (SD 3.6)	2.3 (SD 2.5)	3.0 (SD 4.1)	0.256 [95% CI −0.51; 1.91]
Craniofacial surgery (n, %)				0.201
Fronto-orbital advancement rim	135 (92.5)	52 (91.3)	83 (93.3)	
Reconstruction of one orbit	5 (3.7)	2 (3.8)	3 (3.6)	
Reconstruction of both orbits	127 (94.1)	49 (94.2)	78 (93.9)	
Unknown	3 (2.3)	1 (1.9)	2 (2.5)	
Strip craniotomy	1 (0.7)	0	1 (1.1)	
No surgery	5 (3.4)	4 (7)	1 (1.1)	
Unknown	5 (3.4)	1 (1.7)	4 (4.5)	
Mean age at surgery (m, mean (SD))	11.58 (SD 7.3)	10.49 (SD 3.0)	12.26 (SD 8.8)	0.087 [95% CI −0.78; 4.32]

**Table 2:** Diagnosis in patients with torticollis.

Diagnosis of Torticollis	n = 57 [n, %]
**Incomitant strabismus**	**47 (88.7)**
Pseudo-superior oblique palsy	34 (59.6)
Pseudo-inferior oblique palsy	7 (12.3)
Infantile esotropia and pseudo-superior oblique palsy	3 (5.3)
Fourth nerve palsy	1 (1.8)
Intermittent exotropia and pseudo-inferior oblique palsy	1 (1.8)
Infantile exotropia and pseudo-superior oblique palsy	1 (1.8)
**Concomitant strabismus**	**6 (10.5)**
Accommodative esotropia	3 (5.3)
Intermittent exotropia	2 (3.5)
Micro esotropia	1 (1.8)
**Other**	**1 (1.7)**
Congenital nystagmus	1 (1.8)
**Non-ocular**	**3 (5.3)**

The headings in bold denote the category (along with their respective subcategories) of the ocular diagnosis associated with torticollis.

**Table 3:** Described type of torticollis (n = 57).

Type Torticollis	n = 57 [n, %]
**Face turn**	**16 (28.1)**
Right Left	4 (7.1%) 12 (21.4%)
**Head tilt**	**27 (47.4)**
Right Left	16 (28.6%) 11 (19.6%)
**Combination**	**13 (22.8)**
Turn, chin, tilt Turn, chin Chin, tilt Tilt, turn	4 (7.1%) 3 (5.4%) 2 (3.6%) 4 (7.1%)
**Unspecified**	**1 (1.7)**

The headings in bold denote the category of the type of torticollis (along with the direction in which it occurred).

**Table 4:** Distribution of ophthalmic-related features in the various groups. The number (n) indicated above each feature represents the total count of patients the ophthalmic feature was determined in per group.

Ophthalmic Features	Total [n, %]	Ocular Torticollis [n, %]	No (Ocular) Torticollis [n, %]
**Binocular single vision**	**n = 62**	**n = 19**	**n = 43**
Good Moderate Poor Not present	29 (46.8) 10 (16.1) 11 (17.7) 12 (19.4)	3 (15.8) 3 (15.8) 8 (42.1) 5 (26.3)	26 (60.5) 7 (16.3) 3 (7) 7 (16.3)
**Strabismus**	**n = 145**	**n = 53**	**n = 92**
Esotropia Exotropia ET + vertical XT + vertical Vertical alone Not present	11 (7.6) 8 (5.5) 19 (13.1) 14 (9.7) 17 (11.7) 76 (53.8)	4 (7.5) 2 (3.8) 10 (18.9) 11 (20.8) 13 (24.5) 13 (24.5)	7 (7.6) 6 (6.5) 9 (9.8) 3 (3.3) 4 (4.3) 63 (68.5)
**Ocular motility**	**n = 143**	**n = 53**	**n = 90**
Elevation in adduction RE Elevation in adduction LE Elevation in adduction RLE Depression in adduction RE Depression in adduction LE Depression in adduction RLE Not present	26 (18.2) 20 (14) 23 (16.1) 6 (4.2) 5 (3.5) 2 (1.4) 61 (42.7)	15 (28.3) 12 (22.6) 14 (26.4) 5 (9.4) 2 (3.8) 2 (3.8) 3 (5.7)	11 (12.2) 8 (8.9) 9 (10) 1 (11.1) 3 (3.3) 0 58 (64.4)
**Alphabetical pattern**	**n = 143**	**n = 53**	**n = 90**
V-pattern A-pattern Not present	46 (32.2) 12 (8.4) 85 (59.4)	29 (54.7) 7 (13.2) 17 (32.1)	17 (18.9) 5 (5.6) 67 (75.6)
**Cyclotorsion**	**n = 53**	**n = 30**	**n = 23**
Excyclotorsion Incyclotorsion Not present	33 (62.3) 4 (7.5) 16 (30.2)	22 (73.3)1 (3.3) 7 (23.3)	11 (47.8) 3 (13) 9 (39.1)
Refractive error *	**n = 125**	**n = 45**	**n = 80**
Hypermetropia High hypermetropia Myopia Astigmatism Anisometropia	112 (89.6) 6 (4.8) 2 (1.6) 55 (44) 33 (22.6)	41 (89.1) 2 (4.4) 1 (2.2) 20 (43.5) 12 (26.7)	71 (89.9) 4 (5) 1 (1.3) 35 (44.3) 21 (26.3)
**Amblyopia**	**n = 146**	**n = 54**	**n = 92**
Present Absent	64 (43.8) 82 (56.2)	33 (61.1) 21 (38.9)	31 (33.7) 61 (66.3)

Abbreviations: ET + vertical, esotropia combined with a vertical deviation; XT + vertical, exotropia combined with a vertical deviation; RE, right eye; LE, left eye. * The cumulative percentage exceeds 100% since refractive error can occur combined: spherical (hypermetropia/myopia) and/or astigmatism and/or the presence of an anisometropia.

**Table 5:** Associations between the presence of the investigated ophthalmic features and the presence of torticollis in UCS patients.

Ophthalmic Features	Ocular Torticollis [n, %]	No (Ocular) Torticollis [n, %]	*p*-Value	Cramers’ V
**Binocular single vision**	**n = 19**	**n = 43**		
Present Absent	14 (73.7) 5 (26.3)	36 (83.7) 7 (16.3)	0.277	0.117
**Strabismus**	**n = 53**	**n = 92**		
Present Absent	39 (73.6) 14 (26.4)	29 (31.2) 63 (68.8)	<0.001	0.406
**Ocular motility abnormalities**	**n = 53**	**n = 90**		
Present Absent	50 (94.3) 3 (5.7)	32 (35.6) 58 (64.4)	<0.001	0.574
**Alphabetical pattern**	**n = 53**	**n = 90**		
Present Absent	36 (67.3) 17 (32.7)	22 (24.4) 68 (75.6)	<0.001	0.428
Refractive error *	**n = 45**	**n = 80**		
Hypermetropia Myopia Astigmatism Anisometropia	43 (95.6) 1 (2.2) 20 (44.4) 12 (26.7)	75 (93.8) 1 (1.2) 35 (38.9) 21 (26.2)	1.0 1.0 1.0 1.0	- - - -
**Amblyopia**	**n = 54**	**n = 92**		
Present Absent	33 (61.1) 21 (38.9)	31 (33.7) 61 (66.3)	0.002	0.267

* The cumulative percentage exceeds 100% since refractive error can occur combined: spherical (hypermetropia/myopia), and/or astigmatism, and/or the presence of an anisometropia. In this analysis ‘hypermetropia’ is cumulative of both the patients with ‘hypermetropia’ and ‘high hypermetropia’, and ‘myopia’ is cumulative of both the patients with ‘myopia’ and ‘high myopia’.

**Table 6:** Significant associations between predictor variables for the total group of patients included in the study (n = 146) were found, indicating multicollinearity.

	Presence of Strabismus [*p*-Value, Cramers’ V]	Ocular Motility Abnormalities [*p*-Value, Cramers’ V]	Alphabetical Patterns [*p*-Value, Cramers’ V]
**Presence of strabismus**	-	<0.01 0.483	<0.01 0.416
**Ocular motility abnormalities**	<0.01 0.483	-	<0.01 0.626
**Alphabetical patterns**	<0.01 0.416	<0.01 0.626	-

## Data Availability

The data presented in this study are available on request from the corresponding author. The data are not publicly available due to privacy and ethical reasons.
